# *β*(1,3)-Glucanosyl-Transferase Activity Is Essential for Cell Wall Integrity and Viability of *Schizosaccharomyces pombe*

**DOI:** 10.1371/journal.pone.0014046

**Published:** 2010-11-18

**Authors:** María de Medina-Redondo, Yolanda Arnáiz-Pita, Cécile Clavaud, Thierry Fontaine, Francisco del Rey, Jean Paul Latgé, Carlos R. Vázquez de Aldana

**Affiliations:** 1 Departamento de Microbiología y Genética, Instituto de Microbiología Bioquímica, Consejo Superior de Investigaciones Científicas, Universidad de Salamanca, Salamanca, Spain; 2 Institut Pasteur, Unité des Aspergillus, Paris, France; University College London, United Kingdom

## Abstract

**Background:**

The formation of the cell wall in *Schizosaccharomyces pombe* requires the coordinated activity of enzymes involved in the biosynthesis and modification of β-glucans. The β(1,3)-glucan synthase complex synthesizes linear β(1,3)-glucans, which remain unorganized until they are cross-linked to other β(1,3)-glucans and other cell wall components. Transferases of the GH72 family play important roles in cell wall assembly and its rearrangement in *Saccharomyces cerevisiae* and *Aspergillus fumigatus*. Four genes encoding β(1,3)-glucanosyl-transferases -*gas1^+^*, *gas2^+^*, *gas4^+^* and *gas5^+^*- are present in *S. pombe*, although their function has not been analyzed.

**Methodology/Principal Findings:**

Here, we report the characterization of the catalytic activity of gas1p, gas2p and gas5p together with studies directed to understand their function during vegetative growth. From the functional point of view, gas1p is essential for cell integrity and viability during vegetative growth, since *gas1*Δ mutants can only grow in osmotically supported media, while gas2p and gas5p play a minor role in cell wall construction. From the biochemical point of view, all of them display β(1,3)-glucanosyl-transferase activity, although they differ in their specificity for substrate length, cleavage point and product size. In light of all the above, together with the differences in expression profiles during the life cycle, the *S. pombe* GH72 proteins may accomplish complementary, non-overlapping functions in fission yeast.

**Conclusions/Significance:**

We conclude that β(1,3)-glucanosyl-transferase activity is essential for viability in fission yeast, being required to maintain cell integrity during vegetative growth.

## Introduction

The fission yeast *Schizosaccharomyces pombe* has a rod-like shape and grows asymmetrically at the poles. At the onset of mitosis, polarized growth abates and septum deposition occurs in the middle of the cell, followed by medial fission [1]. The cell wall is an extracellular structure that serves as an exoskeleton for fungi. Its main function is to preserve the osmotic integrity of the cells, but it also contributes to cellular morphology. When the cell wall is removed by lytic enzymes, or in certain cell wall biosynthesis mutants, the cells become ovoid or round, indicating that the cell wall is essential for maintaining cell shape [2], [3]. All the morphogenetic changes, such as tip elongation, septation, mating or sporulation, require continuous cell wall synthesis and remodeling.

In *S. pombe*, the cell wall is composed of mannoproteins (9–14%), α-glucan (18–28%) and β-glucan (46–54%) [4], [5], [6]. β(1,3)-glucan is a major structural component of the fungal cell wall and it forms a fibrillary network, which is thought to be responsible for the mechanical strength of the cell wall. β(1,3)-glucans are present in the inner portion of the wall [7]. The biosynthesis of β(1,3)-glucan is carried out by a β(1,3)-glucan synthase complex, whose catalytic subunit in *S. pombe* is encoded by the *bgs* genes [3], [8]. *S. pombe* contains four proteins of this family; cps1/bgs1p, bgs3p and bgs4p are essential for cell viability during vegetative growth [9], [10], [11], [12], while bgs2p performs an essential role during spore wall formation [13], [14]. It has been postulated that the nascent β(1,3)-glucan chains should be cross-linked to other components of the cell wall by the action of glycoside hydrolases (GH) and transglycosidases [15], [16]. However, *in vivo* evidence of such mechanism has only been shown for the *Saccharomyces cerevisiae* Crh1 and Crh2 proteins, which are involved in the cross-linking of β(1,3)-glucan to chitin [17], [18].

β(1,3)-glucanosyl-transferases of the glycoside hydrolase family 72 (GH72) have been proposed to act remodeling structural β(1,3)-glucans through chain elongation. Originally described in *Aspergillus fumigatus*, this activity has been identified since then in many yeasts [19] and is encoded by the orthologous genes *GEL*, *GAS* and *PHR* in *A. fumigatus, Saccharomyces cerevisiae* and *Candida albicans,* respectively. *S. cerevisiae* contains five genes -*GAS1* to *GAS5*- encoding proteins of the GH72 family [20]. Gas1p is one of the most abundant glycosylphosphatidylinositol (GPI)-anchored cell surface proteins [21], [22]. Gas1p plays an active role in fungal cell wall synthesis during vegetative growth, since *GAS1* deletion results in cells with abnormal morphology and reduced *β*(1,3)-glucan content that is compensated by an increase in chitin and mannan [23], [24], [25]. Recently, it has been described that *GAS2* and *GAS4* are expressed exclusively during sporulation and that the double *gas2 gas4* diploid mutant shows a severe reduction in sporulation efficiency. An analysis of spore ultrastructure indicate that the loss of Gas2p and Gas4p proteins affects the proper attachment of the glucan to the chitosan layer, probably as a consequence of the lack of coherence of the glucan layer [26].

Four genes encoding putative β(1,3)-glucanosyl-transferases -*gas1^+^*, *gas2^+^*, *gas4^+^* and *gas5^+^*- are present in the *S. pombe* genome, suggesting that each protein might perform specific functions at different moments of the life cycle. Indeed, we have recently reported that gas4p is essential for ascospore wall maturation and spore viability in fission yeast [27]. Here we report the characterization of the other three members of the GH72 family in *S. pombe*, which exert their functions during vegetative growth and whose expression is cell-cycle regulated. The three proteins show β(1,3)-glucanosyl-transferase activity with different substrate specificities. Interestingly, *gas1* ^+^ is an essential gene in fission yeast, since *gas1*Δ mutants are unable to survive in the absence of an osmotic stabilizer and undergo cell lysis, mainly during septum degradation.

## Results

### gas1^+^, gas2^+^ and gas5^+^ expression is cell-cycle regulated

*S. pombe* contains four genes encoding proteins belonging to the GH72 family, which contain the conserved transferase domain present in these proteins [19], [28]. In addition to this conserved domain, GH72 proteins have a modular structure and may contain additional domains, such as a Cys-rich region that bears sequence similarity with proteins of the carbohydrate-binding module family 43 (CBM43) [20], [29] and/or a Ser-rich region (Figure S1A). A feature of most of the proteins of this family is the presence of a hydrophobic region at the C-terminus that is part of a GPI attachment site. The gas1p and gas5p proteins contain a putative GPI-anchor site, whereas gas2p apparently lacks the hydrophobic C-terminal region that acts as a signal for this post-translational modification (Figure S1B). To rule out the existence of a frameshift or a sequencing error, the C-terminal region of *gas2^+^* was PCR-amplified from different strain backgrounds and sequenced. The results indicated that the sequence present in the *S. pombe* genome database is correct.

**Figure 1:**
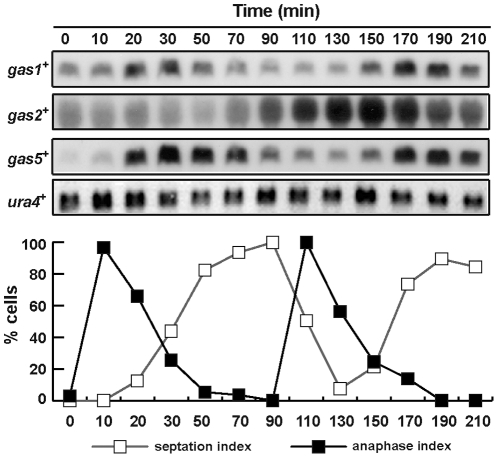
Expression of *gas* ^+^ genes during the cell cycle. Synchrony was induced by arrest-release of a *cdc25-22* mutant and samples were taken at the indicated time-points (minutes). RNA was hybridized with specific probes for each gene. *ura4^+^* was used as a loading control. The graph represents the anaphase or septation index at each time-point.

In agreement with earlier studies [30], Northern analysis indicated that only *gas1^+^*, *gas2^+^* and *gas5^+^* were expressed during vegetative growth and that their expression was periodic along the cell cycle (Fig. 1). Interestingly, the maximum level of expression occurred at different moments. Thus, while *gas1^+^* and *gas5^+^* showed a peak during mitosis, just prior to septation, *gas2^+^* transcript levels were higher during G2. These results suggest that these genes may exert their function at different moments of the vegetative cell cycle.

### gas proteins localize to the cell periphery

**Figure 2:**
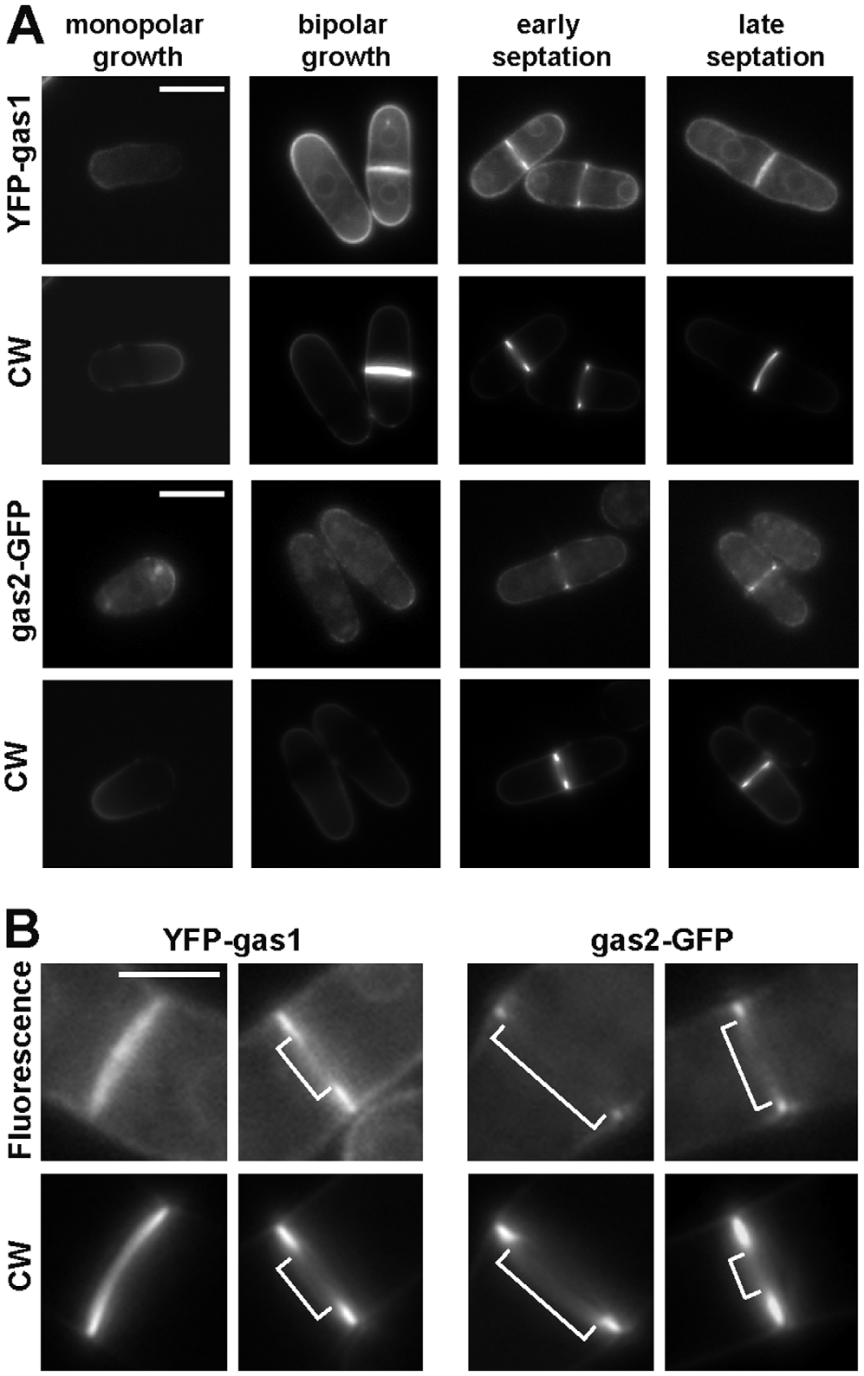
gas1p and gas2p localize to the cell periphery. (A) Localization of YFP-gas1p and gas2-GFP in a wild-type strain. Wild-type cells carrying YFP-gas1p on a plasmid under the control of its own promoter (pMMR18) or carrying gas2p-GFP at the *gas2^+^* locus were grown to early-log phase. Images of cells stained with Calcofluor White (CW) and the fluorescence (YFP-gas1p or gas2-GFP) are shown. Scale bars, 10 µm. (B) gas1p localizes as a disc to the nascent septum, whereas gas2p remains at the septum edging during its synthesis. Scale bars, 5 µm.

In order to determine the localization of gas1p, gas2p and gas5p, the proteins were tagged with fluorescent epitopes. Since the N-terminal signal sequence and the putative C-terminal GPI anchor site could be important for correct localization of the proteins, the epitope was inserted in different regions of the proteins. For *gas1^+^*, the YFP (yellow fluorescent protein) sequence was inserted between the putative signal sequence and the GH72 domain by recombinant PCR and cloned into vector pAL-KS. This plasmid partially suppressed the slow growth rate of *gas1*Δ. The growth rate of the *gas1* null mutant transformed YFP-gas1p was 0.18 h^−1^, compared with a value of 0.25 for the wild-type and 0.05 for the *gas1*Δ. A similar partial complementation has been recently described for *S. cerevisiae* Gas1p-GFP [31]. Since the YFP-gas1p construct was present in a plasmid, we confirmed that the protein was periodically synthesized during the cell by Western analysis, with a pattern similar to the transcription profile of the chromosomal copy (data not shown). Microscopic observation of the transformants revealed that YFP-gas1p localized to the cell periphery and, more precisely, to regions of active growth. The fluorescent signal was clearly observed at the poles of bipolar cells and at the septum of dividing cells (Fig. 2A). However, in monopolar cells the signal was more intense at the non-growing end, perhaps as a remnant of the protein present at the previous septum. For gas5p, a similar strategy was used, since its modular structure was very similar to that of gas1p (an N-terminal signal sequence and a putative C-terminal GPI-anchor site), although no clear localization could be observed. The fluorescence was mainly intracellular, in a vesicular pattern (data not shown), suggesting that the fusion protein had not been correctly processed.

Because *gas2^+^* lacks the putative C-terminal GPI anchor signal, the GFP (green fluorescent protein)-coding sequence was fused in-frame to the last amino acid of gas2p at its chromosomal locus using the cassettes described by Bähler and coworkers [32]. Since *gas2*Δ mutants do not have any clear phenotype (see below), functionality of the gas2p-GFP fusion could not be assessed. gas2p-GFP also localized to the cell periphery, with a localization pattern similar to that of gas1p; i.e., at the tips of bipolar cells and the septum (Fig. 2A). However, gas1p and gas2p localization to the septum region was not identical. gas1p first localized as a ring surrounding the septation region and then rapidly localized as a disc at the nascent septum, which expanded as its synthesis proceeded (Fig. 2B). In contrast, gas2p localized as a ring surrounding the septum and remained inside the cell wall that surrounds the septum: the septum edging (Fig. 2B). Only at later stages, when septum had completely assembled, gas2p could be detected associated with the septum. These results therefore indicate that gas1p and gas2p localize to similar regions of the cell, the growing tips and the septum, even though gas2p lacks a GPI attachment site, and that gas1p and gas2p might play different functions during septum synthesis.

### gas1p is required for correct morphogenesis

**Figure 3:**
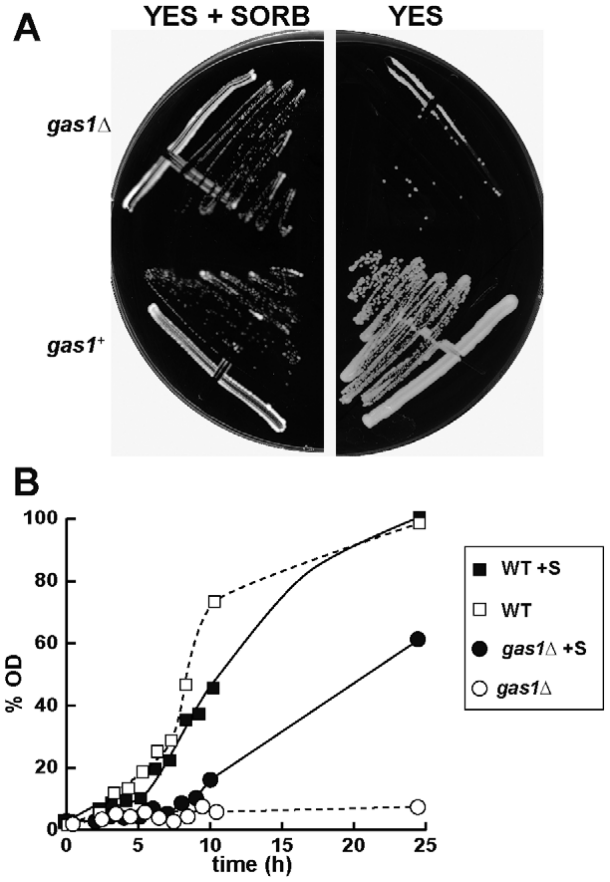
gas1p is essential for cell viability. (A) Growth of wild-type and *gas1*Δ strains in YES (right) or YES+Sorbitol (left) plates. Plates were incubated for 3 days at 32°C. (B) Growth of wild-type and *gas1*Δ strains in liquid medium. Growth rate of *gas1^+^* (WT) and *gas1*Δ strains with (+S) and without sorbitol in the culture medium. Cells were grown overnight in the presence of sorbitol and then diluted with media with or without the osmotic stabilizer. Values are indicated as percentages relative to the final OD of the wild-type strain in sorbitol.

To elucidate whether *gas1^+^*, *gas2^+^* or *gas5^+^* play a role in cell wall remodeling during the cell cycle, mutants lacking each gene were generated with a PCR-based system [33]. Only mutants lacking *gas2^+^* or *gas5^+^* were obtained, suggesting that the third gene might be essential for growth in fission yeast. Accordingly, to confirm this possibility a heterozygous diploid strain -*gas1*Δ::*KanMX4/gas1^+^*- was constructed. Correct deletion of one of the alleles was confirmed by Southern blot, and the heterozygous strain was sporulated and tetrads were dissected on rich medium plates (YES). Tetrad analysis revealed two viable and two unviable spores, all the viable spores being *kan^s^* (data not shown). Microscopic observation of the unviable spores showed that most of them germinated to form two or three rounded cells before growth stopped. When tetrads were dissected on plates containing 1.2 M sorbitol, the spores bearing the *gas1*Δ::*KanMX4* deletion allele were able to germinate and form colonies that were apparently indistinguishable from those of the wild-type strain. *gas1*Δ deletion mutants were only able to grow in the presence of osmotic support (Fig. 3A), thus confirming that *gas1^+^* is an essential gene during vegetative growth in fission yeast. In liquid medium, *gas1*Δ mutant cells grew slower than the wild-type strain in the presence of sorbitol (Fig. 3B) and were unable to grow when transferred to rich or minimal media without an osmotic stabilizer. In contrast, *gas2*Δ mutants and *gas5*Δ mutants, and even the double *gas2*Δ *gas5*Δ mutant, were viable at all temperatures and showed no apparent growth defect in rich medium or minimal medium (data not shown). Taken together, these findings suggest that *gas1^+^*, but not *gas2^+^* or *gas5^+^*, is essential for vegetative growth in absence of osmotic protection.

**Figure 4:**
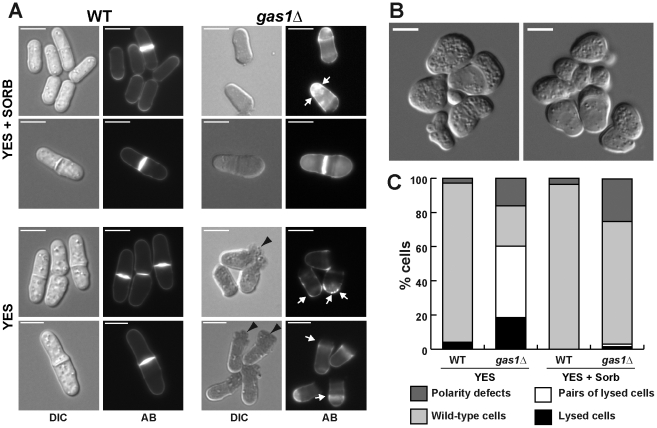
Microscopic appearance of wild-type and *gas1*Δ mutant cells. (A) The wild-type and the *gas1*Δ strains were incubated in rich media with or without osmotic support. Samples were directly stained with aniline blue before photographs were taken. Differential interference contrast (DIC) or fluorescent (AB) images are shown. White arrows indicate an abnormal deposition of glucan-like cell wall material; black arrowheads indicate sites of cell lysis. Bars, 10 µm. (B) *gas1*Δ polarity defects in the absence of osmotic support. Images illustrating the polarity defects are shown. Bars, 10 µm. (C) Percentage of *gas1*Δ cells showing polarity or lysis defects with and without osmotic support. Wild-type and *gas1*Δ cells that had been growing overnight in rich media with sorbitol were diluted in fresh media with and without 1.2 M sorbitol and incubated at 32°C for 2 hours before the percentage of isolated lysed cells, pairs of lysed cells, wild-type cells, and cells with polarity defects was calculated.

The morphology of mutant cells was also analyzed during growth at different temperatures by microscopic observation using aniline blue to stain the *β*(1,3)-glucan of the cell wall [34]. *gas1*Δ cells grown on plates with sorbitol were shorter and rounder than the wild-type, and some lysed cells were also observed (Fig. 4A). In some cases, cell wall staining was not uniform, an abnormal deposition of cell wall material that stained more intensely with aniline blue being observed (Fig. 4A, arrows). When transferred to media without an osmotic stabilizer for two hours, a large fraction of the *gas1*Δ mutant cells (around 60%) underwent lysis. Of the surviving cells, half of them had lost their polarity and were rounded, with large accumulations of abnormal cell wall material that were even visible with Nomarsky optics (Fig. 4B and C). Interestingly, although in some cases we observed lysis at the cell tips, many lysed cells displayed a similar terminal phenotype, appearing as pairs of lysed cells, as if they had undergone lysis during the cell separation process (Fig. 4A and C). To confirm this hypothesis, a time-lapse experiment was performed to monitor the growth over time of a *gas1*Δ mutant. When *gas1*Δ cells were inoculated in rich media without sorbitol, cells were able to grow normally, although some of them displayed polarity defects, and they were also able to assemble the separation septum properly (Figure S2). However, when cell separation started and the newly generated ends started to appear round, lysis occurred suddenly (minutes 19, 27, 80, 98). These results suggest that *gas1^+^* is essential for the maintenance of cellular integrity and viability during vegetative growth and also for cell morphogenesis. In the absence of gas1p, cell lysis takes place in actively growing regions (poles and septum), and cells that do not lyse display morphogenetic defects, manifested as widened poles or widened medial regions (Figure S2, asterisks).

### gas1p, gas2p and gas5p display β(1,3)-glucanosyl-transferase activity

**Figure 5:**
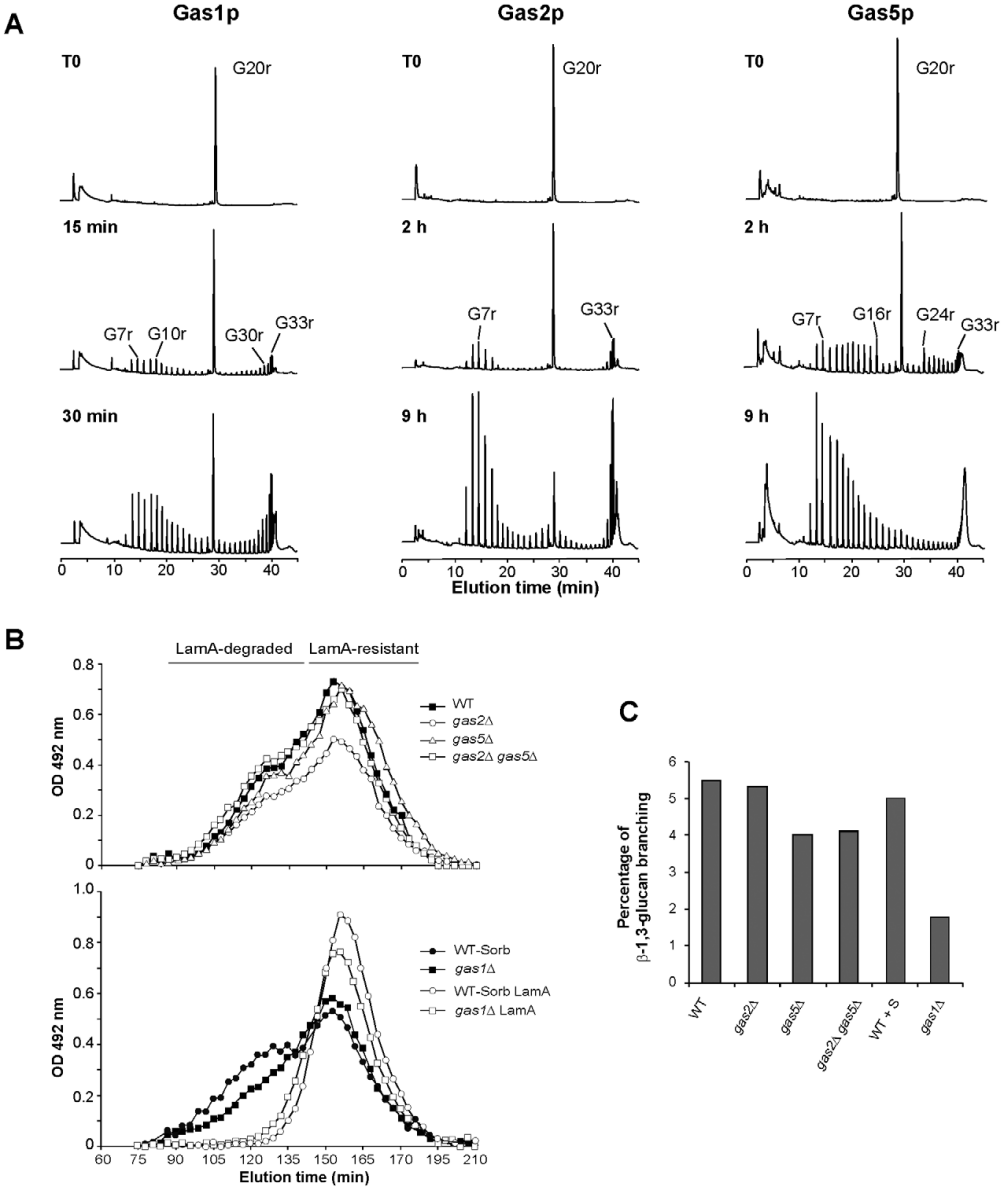
Cell wall composition of *gas*Δ mutants and analysis of enzymatic activity. (A) Fission yeast gas proteins display β(1,3)-glucanosyl-transferase activity. Recombinant gas1p, gas5p and gas2p expressed *P. pastoris* were incubated with G20r in 50 mM acetate buffer (pH 5.0) for the indicated times. The reaction products were analyzed by HPAEC-PED on a CarboPAC-PA200 column. The main reaction products are indicated. (B) For the preparation of cell walls, strains h20 (WT), YMMR16 (*gas2*Δ), YMMR32 (*gas5*Δ) and YMMR40 (*gas2*Δ *gas5*Δ) were grown in YES medium. YMMR106 (*gas1*Δ) was grown in YES medium supplemented with 1.2 M sorbitol (+S) and compared with a wild-type strain (h116) grown under the same conditions. Gel filtration chromatography of carboxymethylated cell wall fractions on an HR500S column with or without digestion with Laminarinase A (LamA). Sugars were detected by the phenol sulphuric method. (C) Percentage of β(1,3)-glucan branching in cell wall fractions of *gas*Δ mutants. The amount of branching was estimated after laminarinase A digestion and HPAEC analysis.

The similarity between *S. pombe* gas proteins and *S. cerevisiae* Gas1p or *A. fumigatus* Gel1p, and the sequence conservation around the two aspartic acids that form the catalytic pair -FF(A/S)GNE*V (the acid-base donor) and F(F/L)SE*(Y/F)GCN (the nucleophilic residue) [35], [36]- suggest that gas1p, gas2p and gas5p could display the same glucanosyl-transferase activity as other GH72 proteins [20], [27], [28], [36]. To test this notion, recombinant versions of gas1p and gas5p (lacking the GPI moiety and containing 6 His at the C-terminus: r-gas1p and r-gas5p) and gas2p (containing two sets of 6 His, one before the GH72 domain and another at the C-terminus- r-gas2p) were analyzed. The purified proteins were incubated with reduced laminari-oligosaccharides of 13 or 20 glucose residues (G_13r_ and G_20r_) for different times, and the products of the reaction were analyzed by high-performance anion exchange chromatography coupled to pulse electrochemical detection (HPAEC-PED) (Fig. 5A and Figure S3B). The resulting products were smaller and larger than the starting substrate, indicating that all enzymes were catalyzing a transfer reaction. The results revealed that the products of r-gas1p were very similar to those generated by *Sc*Gas1p, *Sc*Gas4p, *Af*Gel1p, *Af*Gel2p or *Sp*gas4p [19], [20], [27], [28], [37].

The chromatogram shows that the enzyme preferentially released an oligosaccharide of 6-8 glucose units from the reducing end of G_13r_ and transferred it to the acceptor laminari-oligosaccharide (Figure S3A). Thus, the major initial products were G_6–8r_ and rG_18–20r_, in agreement with the two-step reaction scheme previously described by Hartland et al. [38]. Analysis of r-gas2p and r-gas5p indicated a slight difference with r-gas1p activity. Mainly, G_13r_ was a poor substrate for r-gas2p (Figure S3B), which only weakly transferred glucan fragments smaller than 10 glucose residues (Fig. 5A), while gas5p catalyzed the transfer of a glucan with a degree of polymerization ≥4 (Fig. 5A and Figure S3A). These results indicated that although the catalytic reaction was the same, the three enzymes had slight differences in substrate recognition. In all cases, the pattern observed after prolonged incubation times indicated that the initial transfer products could be subsequently re-used either as donors or acceptors, resulting in a broad range of transfer products of increasing size (degree of polymerization >30) until they became water-insoluble. The minimum oligosaccharide that could be used in the reaction was an oligosaccharide of 9 glucose units (data not shown), and the optimum pH was slightly acid: pH 5.0. In light of the foregoing, we conclude that gas1p, gas2p and gas5p are true *β*(1,3)-glucanosyl-transferases *in vitro*, but that the three enzymes differ in their affinities for different substrates, the cleavage point, and also in their transfer products.

### Cell wall composition of gasΔ mutants

To test whether GH72 proteins had any role in cell wall construction *in vivo* in fission yeast, the cell wall composition of the different mutants was analyzed. Cell walls were purified and fractionated in alkali-soluble (AS) and alkali-insoluble (AI) fractions. The major cell wall fraction was the AS. Total carbohydrate analysis showed no quantitative differences in the AS fraction of all strains (Figure S3B). The AS fraction was partially digested by treatment with endo-β(1,3)-glucanase (Laminarinase A, LamA). Gel filtration of the AS fractions carboxymethylated before or after LamA treatment indicated that β(1,3)-glucans corresponded to the fractions with the highest molecular weight. The LamA resistant fraction eluted later (Fig. 5B). This fraction most probably contained the α (1,3)-glucans, which are the other major cell wall component resistant to endo-β-1,3 glucanase. The same digestion and elution profiles were seen for the AS fraction from the wild-type strain and the *gas2*Δ, *gas5*Δ and *gas2*Δ *gas5*Δ mutants (Fig. 5B). However, the AS fraction from the *gas1*Δ mutant grown in medium containing 1.2 M sorbitol had a lower amount of the high molecular weight β(1,3)-glucans, as compared to the wild-type strain grown under the same conditions. It has recently been described that LamA digestion and HPAEC analysis allows to estimate the level of β(1,6)-branching of the cell wall β(1,3)-glucans [39]. As shown in Fig. 5C, a low percentage of branching was observed in *gas5*Δ and *gas2*Δ *gas5*Δ mutants and the decrease was even more pronounced in *gas1*Δ cell walls. No differences were observed for the *gas2*Δ mutant. The differences in cell wall composition in the *gas1*Δ mutant are also in good agreement with the high degree of staining with aniline blue (Fig. 4), a dye that gives stronger fluorescence with linear β(1,3)-glucan chains than with β(1,3)/β(1,6)-glucans.

For the alkali-insoluble fraction, only a significant amount could be recovered and analyzed in the *gas1*Δ mutant and in the double *gas2*Δ *gas5*Δ mutant in comparison with the other strains (Figure S3B). The AI fraction was however very limited, since it did not exceed 8% of the AS fraction. This AI fraction did not contain GlcNAc (data not shown). Gel filtration analysis of the carboxymethylated AI polysaccharides revealed that the LamA digested material of the *gas1*Δ and the double *gas2*Δ *gas5*Δ mutants contained the largest glucans (Fig. 5C). Thus, the overall structural organization of the cell wall of the *gas2*Δ *gas5*Δ and *gas1*Δ mutants were significantly modified from that of the wild type. The reduction in the size of the β(1,3)-glucan chains of the major cell wall fraction seen in the *gas1*Δ mutant is in agreement with the biochemical function of the gas1protein.

### β(1,3)-glucanosyl-transferase activity is essential during vegetative growth

**Figure 6:**
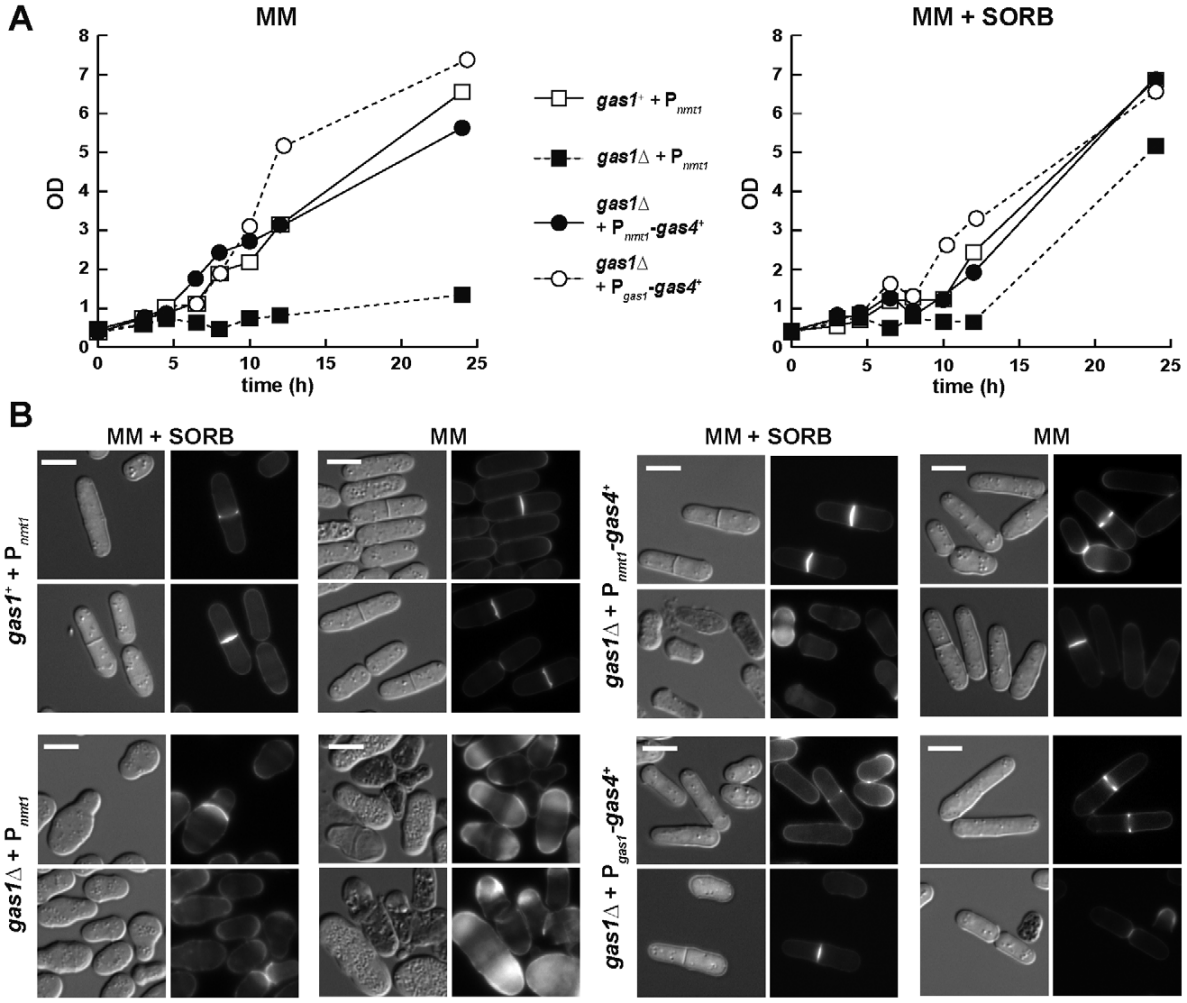
Ectopically expressed gas4p complements the unviability defects of *S. pombe gas1*Δ mutant. (A) Growth rate of the *gas1^+^* strain carrying vector (YMMR135) and *gas1*Δ cells harboring vector (YMMR133), P*_nmt1_*-*gas4^+^* (YMMR134) or P*_gas1_*-*gas4^+^* (YMMR140), incubated with (right) and without (left) sorbitol in the culture medium. (B) Microscopic appearance of *gas1*Δ cells ectopically expressing *gas4^+^*. Wild-type cells and *gas1*Δ strains harbouring P*_nmt1_*-*gas4^+^* or P*_gas1_*-*gas4^+^* were incubated in minimal media with or without osmotic support. Samples were stained with aniline blue before images were captured. Differential interference contrast (DIC) or fluorescent (AB) photographs are shown. Bars, 10 µm.

We have previously shown that another member of this family, gas4p, is essential for spore wall assembly and that it catalyzes a transfer reaction with similar specificity to that found for gas1p [27]. We therefore wondered whether gas4p could complement the cell lysis phenotype and/or the morphogenetic defects of a *gas1*Δ mutant. In order to allow the expression of *gas4^+^* during mitotic growth, the coding region was cloned under the control of the *nmt1^+^* promoter, which is induced in the absence of thiamine. Since *gas1^+^* is periodically expressed during the cell cycle, *gas4^+^* was also placed under the control of the *gas1^+^* promoter (P*_gas1_*) to allow its cell cycle-regulated expression. Strains *gas1*Δ and the wild-type carrying the vector were used as controls to analyze growth in liquid media, with or without osmotic support. *gas1*Δ cells carrying the vector were able to grow in the presence of sorbitol, but not when the osmotic stabilizer was absent (Fig. 6A). However, overexpression of *gas4* ^+^ under the control of the *nmt1* promoter or the *gas1* ^+^ promoter was able to complement the growth defect of *gas1*Δ cells without osmotic support, growth rates similar to those of wild-type cells being observed.

**Figure 7:**
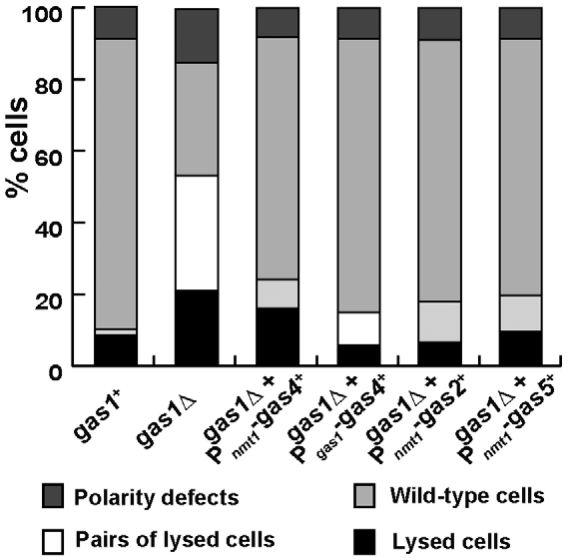
Over-expression of *gas* ^+^ genes complements the defects of the *gas1*Δ mutant. Percentage of *gas1*Δ cells showing polarity or lytic defects with and without osmotic support. Wild-type and *gas1*Δ cells overexpressing *gas2* ^+^, *gas4* ^+^ or *gas5* ^+^ that had been growing overnight in minimal media with sorbitol were inoculated in fresh media with and without 1.2 M sorbitol and incubated at 32°C for 4 hours before the percentage of isolated lysed cells, pairs of lysed cells, healthy isolated cells, and cells with polarity defects were calculated.

To test whether the morphogenetic defects of *gas1*Δ cells were also complemented by *gas4* ^+^, the morphology of the transformants was analyzed and the different defects were quantified. Similar to what happened in rich media without osmotic support, *gas1*Δ cells harboring the vector were rounder and shorter than the wild-type (Fig. 6B), and around half of the cells underwent lysis in these culture conditions (Fig. 7). In contrast, cells ectopically expressing *gas4^+^* (under the control of P*_nmt1_* or P*_gas1_*) were similar to the wild-type strain (Fig. 6B) and the number of dead cells was severely reduced with respect to *gas1*Δ cells (Fig. 7). Thus, gas4p is able to fully complement the defects of *gas1*Δ cells. Interestingly, the P*_gas1_*-*gas4^+^* construct was more efficient at reverting *gas1*Δ unviability than P*_nmt1_*-*gas4^+^*, suggesting that proper temporal expression is important for better complementation.

In the *gas1*Δ mutant strain, gas2p and gas5p are present although they are not able to compensate the loss of gas1p. To test whether higher levels of gas2p and gas5p than those present in vegetative cells would be able to revert the *gas1*Δ phenotype, *gas2^+^* and *gas5^+^* were also cloned under the control of the *nmt1* ^+^ promoter and introduced in *gas1*Δ haploid cells. Interestingly, overexpression of these two genes also complemented the lysis and morphogenetic defects of *gas1*Δ cells (Figure S4 and Fig. 7). Together, these results indicate that a certain level of β(1,3)-glucanosyl-transferase activity is required for proper morphogenesis and viability in fission yeast, regardless of the length of the transferred oligosaccharide.

## Discussion

Proteins of the GH72 family display glucanosyl-transferase activity. They are thought to be involved in β(1,3)-glucan remodeling and this activity is of great importance in fungal biology [16]. *In S. pombe*, 4 genes encoding GH72 proteins have been identified. We have previously characterized the gas4p function in *S. pombe* as a glucanosyl-transferase necessary for spore wall assembly and spore viability [27]. The other three genes are expressed during vegetative growth and their functions were investigated in this study.

The biochemical activity of GH72 proteins was first demonstrated for *Af*Gel1p, and this was reported to catalyze *in vitro* a two-step β(1,3)-glucanosyl transferase reaction: first, an internal glycosidic linkage of a donor β(1,3)-glucan chain is cleaved and the new reducing end is transferred to the non-reducing end of an acceptor β(1,3)-glucan chain through a β(1,3)-linkage, resulting in its elongation [19], [38]. Our results indicate that *S. pombe* gas1p, gas2p and gas5p are also endowed with the same β(1,3)-glucanosyl transferase activity. Differences between these proteins were observed in the pattern of the products released. Recombinant gas1p displayed the same β(1,3)-glucanosyl transferase activity as those described for *Sc*Gas1p, *Sc*Gas4p, *Af*Gel1p, *Af*Gel2p and *Sp*gas4p [19], [20], [27], [28], [37], catalyzing the transfer of an oligosaccharide of 6-8 glucose units, while the activity of gas5p was similar to that described for *Sc*Gas5p [20], transferring oligosaccharides of more than 4 monomers between glucan molecules. Finally, gas2p preferentially used longer oligosaccharides as substrates (G_20r_ *vs.* G_13r_) and the oligosaccharides transferred from the donor substrate to the acceptor molecule were also longer.

All yeasts and fungal species sequenced so far have members of this family in their genomes, and they usually form a redundant family of enzymes [20]. Molecular and biochemical analysis of gas1p, gas2p and gas5p during fission yeast vegetative growth has revealed similarities and differences with *S. cerevisiae,* possibly reflecting differences in the biology or variations in cell wall composition between these two yeasts. In spite of their homologous sequences and the similar enzymatic activities of the encoded proteins, disruption of these three genes resulted in very different phenotypes in *S. pombe*: *gas2*Δ and *gas5*Δ mutants had a phenotype similar to that of wild-type cells, whereas *gas1*Δ mutant cells were only viable in the presence of an osmotic supplement, indicating that gas1p is essential for viability in fission yeasts. This difference in the requirements for Gas proteins during vegetative growth is similar to the situation described for *S. cerevisiae,* in which Gas1p plays a much more relevant role in cell wall construction than Gas5p and Gas3p [20], [26], or for *A. fumigatus*, where *gel1*Δ mutants have no apparent phenotype, while *gel2*Δ mutants have severe growth defects and abnormal mycelial morphology and *GEL4* is essential [37], or for *C. albicans*, where *PHR1 and PHR2* genes are differentially expressed depending on the extracellular pH [40], [41], [42].

A major result of this study is that the β(1,3)-glucanosyl transferase gas1p of *S. pombe* is an essential protein for vegetative growth in fission yeast. The structural organization of the β(1,3)-glucan of the cell wall is clearly altered in *gas1*Δ cells. Disruption of *gas1* resulted in an abnormal β-glucan deposition and a reduction of the size of β(1,3)-glucan chains in the cell wall of *S. pombe,* in agreement with the biochemical function of these proteins. In *S. cerevisiae*, deletion of *GAS1* also affects cell wall composition [21]. *gas1*Δ mutants have a decrease in β(1,3)-glucan levels that is compensated by an increase in mannan and chitin contents [24], [25], [43], [44]. An interesting observation is that a decrease in β-glucan branching has also been observed in other *gas* mutants. A similar result was found for the *S. cerevisiae gas1*Δ mutant (Aimanianda et al., personal communication). Since no branching activity has been detected in any of the GH72 members *in vitro*, these observations suggested that the decrease in branching is a compensatory reaction to the Gas1 deletion as it has been seen also for chitin in other yeasts [25]. It also suggests that the synthase, the branching enzymes and the elongases could interact in the remodeling of the cell wall glucans. No such complex has yet been identified in fungi. This is the next challenge in the cell wall field and the data presented in this study show that *S. pombe* is an interesting model for understanding β-glucan synthesis and remodeling.

## Materials and Methods

### Strains and growth conditions

The *S. pombe* strains used in this study are listed in Table 1. Yeast cells were grown on YES media or minimal media (EMM) with appropriate supplements [45]. When required, sorbitol was added at final concentration 1.2 M. Synchronization of strains carrying the thermosensitive *cdc25-22* mutation was achieved by growing the cells at 25°C to the early log phase (OD_595_ = 0.5) and then shifting the cultures to 37°C for 4 hours. Cells were released from arrest by transfer to 25°C, and samples were taken every 20 minutes.

**Table 1:** Yeast strains used in this study.

Strain	Genotype	Source
h20	h^−^ *leu1-32*	Lab stock
h116	h^+^ *leu1-32 ura4-*Δ*18 ade6-M216*	Lab stock
h54	h^−^ *leu1-32 ura4-*Δ*18 ade6-M210*	Lab stock
YMMR16	h^−^ *leu1-32 gas2*Δ*::kanMX4*	This study
YMMR17	h^−^ *leu1-32 gas4*Δ*::kanMX4*	This study
YMMR32	h^+^ *leu1-32 ura4-*Δ*18 ade6-M216 gas5*Δ*::kanMX4*	This study
YMMR37	h^+^ *leu1-32 ura4-*Δ*18 ade6-M216 gas5*Δ*::kanMX4 + pMMR18*	This study
YMMR40	h^−^ *leu1-32 gas2*Δ*::kanMX4 gas5*Δ*::kanMX4* (YMMR16× YMMR32)	This study
YMMR41	h^−^ *leu1-32 ade6M-216 gas2*Δ*::kanMX4 gas5*Δ*::kanMX4* (YMMR16× YMMR32)	This study
YMMR104	h^+^/h^−^ *leu1-32/leu1-32 ura4-*Δ*18/ura4-*Δ*18 ade6-M120/ade6-M216 gas1*Δ*::kanMX4/gas1^+^*	This study
YMMR105	h^−^ *leu1-32 gas2-GFP*	This study
YMMR106	h^+^ *leu1-32 ura4-*Δ*18 ade6-M216 gas1*Δ*::kanMX4*	This study
YMMR133	h^+^ *leu1-32 ura4-*Δ*18 ade6-M216 gas1*Δ*::kanMX4* + pC530	This study
YMMR134	h^+^ *leu1-32 ura4-*Δ*18 ade6-M216gas1*Δ*::kanMX4* + pMMR39	This study
YMMR135	h^+^ *leu1-32 ura4-*Δ*18 ade6-M216* + pC530	This study
YMMR138	h^+^ *leu1-32 ura4-*Δ*18 ade6-M216 gas1*Δ*::kanMX4 +* pMMR41	This study
YMMR139	h^+^ *leu1-32 ura4-*Δ*18 ade6-M216 gas1*Δ*::kanMX4* + pMMR42	This study
YMMR140	h^+^ *leu1-32 ura4-*Δ*18 ade6-M216 gas1*Δ*::kanMX4* + pMMR43	This study

### Plasmid constructions and DNA manipulations

The oligonucleotides used in this study are listed in Table 2. Construction of plasmid pMMR13 carrying the *gas1^+^* coding sequence under its own promoter was achieved by PCR amplification of the coding sequence using oligonucleotides 1076 and 1032 that introduced *BamH*I and *Xho*I sites at the ends. The sequence was then cloned between the *Bam*HI and *Xho*I sites of the pAL-KS vector. Plasmid pMMR39 (P3x*_nmt1_*-*gas4^+^*) was constructed by PCR amplification of the coding sequence of *gas4^+^* using primers 1427 and 1428 that introduced *Bam*HI and *Xho*I at the ends, and cloning the resulting fragment between the *Bam*HI and *Xho*I of the pJCR1-3XL vector [46]. The *gas4^+^* coding sequence was placed under the control of *gas1^+^* promoter (P*_gas1_*) by PCR amplification of the *gas1^+^* promoter sequence with oligonucleotides 1431 and 1432 that introduced *Sph*I and *Xho*I sites at the ends, and cloning the resulting fragment between the same sites of plasmid pMMR39 to generate plasmid pMMR43. Construction of plasmids pMMR41 (P3x*_nmt1_*-*gas2^+^*) and pMMR42 (P3x*_nmt1_*-*gas5^+^*) was achieved by PCR amplification of the coding sequence of *gas2^+^* and *gas5^+^* using specific primers 1433–1434 (that introduced *Xho*I and *Bam*HI at the ends), and 1435–1436 (that created *Xho*I and *Not*I sites), respectively. These fragments were cloned into the pJCR1-3XL vector.

**Table 2:** Oligonucleotides used in this study.

Name	SEQUENCE
998	CTTCCACTGGTGAGCGTGTCTT
999	ACCATGATCCTGAGTGTAAGCGTT
1000	ACTTCTCTTCCCCCTACTCCTTCA
1001	AGCACCGCTGCTGCTCTTAG
1004	TGCTAACTATACTGGAGACGGTGATT
1005	AAGCGAGAAAGGTTAAAAGTAGAAGC
1025	TAAAGCGGCCGCTCATTCGTCATACTGTATTACGTTGG
1026	GGGGATCCGTCGACCTGCAGCGTACGTAAATTTGGTAAAGGAGACCATG
1027	AACGAGCTCGAATTCATCGATGATTGTTGGTGAGCTTAAGGCATAATTTCC
1028	TAAAACTAGTATTCGCTGATTGAATGTCCATATAAG
1032	TAAACTCGAGGAAACGAAGCACCCCATACTTGTC
1047	TAAATCTAGAACCGCTACCATATATAGTGGGATAGC
1048	GGGGATCCGTCGACCTGCAGCGTACGAGGAAGTTCATAGCAGATATAGGTTC
1049	AACGAGCTCGAATTCATCGATGATGGTATACTGGCAATTAGCGGACTTG
1050	TAAACTCGAGCGAATATATCAATAATGAACTTGAGAAG
1076	GGATCCTCAAGCTTGCTTCTTACATTGCAT
1140	CCAGTGAAAAGTTCTTCTCCTTTACTGTTAATTAAAGGAGAAACTGAGGCTTTAGCGAGGC
1141	GGGATTACACATGGCATGGATGAACTATACAAAGTTCACGTTGATGGTCGTTACTTT
1142	AGTAGGATCAGAGCTTGAAATGGC
1259	GAAGCTGAATTCTCAGTTTCTCTTGTTCACGTTGATGGT
1260	GCTGGCGGCCGCGCTGCTCTTAGAGCTGGAGTTGCT
1263	GAAGCTGCAGGAGACTCTGCCTCTTCCGCTATCAAA
1264	TTGTTCTAGAATGGAAGAAGTAGCCGAGGAGCTTCC
1353	ATTAGCGGCCGCGGTCTCGTTGGCCTCGCTAAA
1354	GGGGATCCGTCGACCTGCAGCGTAGTTATCATAGGGAGCCAAAGAATCAAC
1355	AACGAGCTCGAATTCATCGATGATCTAGTGCTTCTTATGAATCGACTATGTC
1356	ATAATGCGGCCGCTGCTGCTGCCAGAGCTGTTGC
1371	TACACTCAGGATCATGGTGCCGATTCTTGTAGCTGGGGTGGTGTTGGTGAGCTTAAGGCACGGATCCCCGGGTTAATTAA
1372	TTACAATAGTACAGGTGGCTGATTACAAACTCATGGATTATATGCAAGAATGGTATGTGACGAATTCGAGCTCGTTTAAAC
1386	TTGTTCTAGAATGCTACAAGAATCGGCACCATG
1395	GAAGCTGAATTCCATCATCATCATCATCATTATTTCGAGCCTTTGACTATCAAGGGT
1427	AGCTCTCGAGGCATTCTTGGAGAAGGTATTGAATATTATATTGC
1428	AGCTGGATCCCATTCAGCTAAGCTCTAAATTTCAACAATTC
1431	CTCGCATGCTCAAGCTTGCTTCTTACATTGCA
1432	TCCCTCGAGGTAAACAAGAAACAGGGTATTAAAAG
1433	AGCTCTCGAGTATAAATACCAGTCGCCACTCTTTATTAAC
1434	AGCTGGATCCATGAGGAAATTATGCCTTAAGCTCACCAAC
1435	AGCTCTCGAGCCTCGAACTGAACCTATATCTGCTATG
1436	AGCTGCGGCCGCTTACGAAAAATGAAAGCGAGTGGTATG

### Construction of null mutants and strains

The coding sequence of *gas1^+^* corresponding to GH72 domain was replaced by the *KanMX4* cassette using the recombinant PCR approach described by Wach [33]. For this purpose, DNA fragments corresponding to the 5′ and 3′ regions were PCR-amplified using oligonucleotides 1353–1354 and 1355–1356, respectively. The resulting fragments were fused by recombinant PCR to the *KanMX4* cassette from plasmid pFA6a-*KanMX4* [47]. The entire coding sequences of *gas2^+^* and *gas5^+^* were deleted to create the null mutants by replacing them by the *KanMX4* cassette using the same approach. For *gas2^+^*, oligonucleotides 1025, 1026, 1027 and 1028 were used; for *gas5^+^*, the cassette was constructed using oligonucleotides 1047, 1048, 1049 and 1050. The N-terminally YFP-tagged *gas1^+^* fusion was constructed by PCR amplification of 570 and 210 bp DNA fragments with oligonucleotides pairs 1076–1140 and 1141–1142, respectively. The resulting fragments were fused by recombinant PCR to the YFP coding sequence obtained from plasmid pBS7 (Yeast Resource Center). The amplified fragment contained the YFP coding region fused in-frame to the 23th codon of the *gas1^+^* gene and was then cloned at the *Hind*III sites of plasmid pMMR13, generating pMMR18. For C-terminal tagging of gas2p with GFP, the pFA6a-GFP(S65T)-*KanMX6* plasmid [32] was used as a template to amplify the GFP-*KanMX6* cassette flanked by *gas2^+^* homology regions with oligonucleotides 1371 and 1372.

### RNA isolation and Northern blot analyses

Total RNA was prepared from 1.3×10^9^ cells using the method described previously [27]. For Northern blot analyses, 12.5 µg of RNA was used. The DNA probes used were 400–500 bp internal fragments amplified by PCR with oligonucleotides 1000–1001 for *gas1^+^*, 998–999 for *gas2^+^*and 1004–1005 for *gas5^+^*, or a 1.7 kb *Bam*HI-*Hin*dIII fragment obtained from plasmid pSK-*ura4* ^+^ for *ura4* ^+^. Probes were labeled using the random priming method.

### Microscopy techniques

For light microscopy, the β(1,3)-glucan of the cell wall was stained with aniline blue [34]. Samples were viewed using a Leica DMRXA microscope equipped for bright-field and Nomarski optics and epifluorescence and photographed with a Leica DFC350FX camera or an ORCA-ER camera. To determine the percentage of cell lysis, cells were stained with 0.6% methylene blue. Dead cells accumulate the dye and appear dark on a phase contrast microscope.

### Cell wall analysis

Fractionation of cell wall polysaccharides was achieved as follows. A portion of 200 mg of dry cell walls was treated twice with 10 ml of 25 mM Tris-HCl, 5 mM EDTA, pH 7.4, 2% SDS and 40 mM β-mercaptoethanol at 100°C for 10 minutes. Insoluble materials were recovered by centrifugation (10 minutes, 3000 g) and washed extensively with water. Alkali-soluble materials were extracted twice with 1M NaOH at 65°C for 1h. Alkali-insoluble materials were removed by centrifugation (10 minutes, 3000 g) and washed extensively with water. Alkali-soluble fractions were neutralized by the addition of acetic acid and then freeze-dried. Precipitates were recovered by centrifugation (10 minutes, 3000 g), dialyzed against water, and freeze-dried. Protein amounts were estimated with the BCA reagent (Pierce). Total hexose contents were estimated using the phenolsulfuric acid method [48]. Determination of α (1,3)-glucan and β(1,3)-glucan content was achieved by digestion of the cell wall material with specific glucanases (enzymatic unit: 1 µmol Glc equiv/min). In the case of β(1,3)-glucan, 20 µl of cell wall extracts (5 mg/ml) was incubated in 100 µl of 100 mM NaOAc, pH 6.2, containing 0.4 units of laminarinase A (a recombinant endo-β(1,3)-glucanase from *Thermotoga neapolitana*, expressed in *E. coli*, and a kind gift from Dr Vladimir V. Zverlov, Institute of Molecular Genetics, Moscow, Russian Federation) at 37°C for 6h [49]. Glucan contents were estimated as the amount of reducing sugars released in each treatment by the PAHBAH method [38].

### Estimation of β(1,3)-glucan branching

The branching point of β(1,3)-glucan is resistant to laminarinase A digestion, resulting in a specific trisaccharide that can be measured by HPAEC. Cell wall fractions were subjected to laminarinase A digestion as described above and the products were analyzed with HPAEC on a CarboPAC-PA1 column (4.6×250 mm, Dionex), as described [39]. Branching was correlated to the presence of a specific peak absent when a linear β(1,3)-glucan was digested.

### Gel filtration chromatography

The molecular size of cell wall polysaccharides was estimated by gel filtration chromatography. Cell wall fractions were carboxymethylated to solubilise the polymers. 10 to 20 mg of cell walls was suspended in 1 ml of 1.6 N NaOH. After the addition of 1 ml monochloroacetic acid (MCA, 0.3 mg/ml, pH 10–11 by addition of NaOH), samples were incubated at 75°C for 5h. Then, another 2 ml of MCA were added and the samples were incubated at 75°C overnight. After neutralization by the addition of acetic acid, samples were dialyzed against water and freeze-dried. Carboxymethylated fractions were dissolved in 50 mM NaOH, and 250 mM AcONa at a final concentration of 10 mg/ml, and 300 µl were loaded onto a HR500S gel filtration column (GE Healthcare, 30×1.6 cm) and eluted at a flow rate of 0.3 ml/min. Sugars were detected with the phenolsulphuric acid method [48].

### Expression of recombinant gas proteins in *Pichia pastoris*

*P. pastoris* KM71H (Invitrogen) and the expression vectors pPICZαA and pPICZαB were used to express recombinant proteins. Truncated forms of *gas1^+^* and *gas5^+^* were generated by PCR-amplification of the gene with specific oligonucleotide pairs. *gas1^+^* was amplified with primers 1259 and 1260, which created *Eco*RI and *Not*I sites at the ends. For *gas5^+^*, the primers were 1263 and 1264, which incorporated *Pst*I and *Xba*I sites at the ends. The *gas1^+^* PCR product was digested by *Eco*RI and *Not*I and cloned into the vector pPICZαA, generating plasmid pMMR22. The *gas5^+^* PCR product was digested with *Pst*I and *Xba*I and cloned into the pPICZαB vector to obtain plasmid pMMR24. A *gas2^+^* recombinant form lacking the last 27 nucleotides was generated by PCR amplification of the gene with oligonucleotides 1395 and 1386. The forward primer introduced a 6xHis coding sequence and incorporated an *Eco*RI site at the 5′ end. The reverse primer created a *Xba*I site at the 3′ end. The PCR product was digested by *Eco*RI and *Xba*I and cloned into pPICZαA, generating plasmid pMMR35. The transformation and production of the recombinant proteins was performed according to manufacturer's instructions (Invitrogen).

### Enzymatic analysis of recombinant proteins

Culture filtrates were concentrated using Amicon Ultra Centrifugation Devices (Amicon) and dialyzed against Bind-Buffer (50 mM NaH_2_PO_4_, pH 8.0, 300 mM NaCl, 10 mM imidazole). Then, they were incubated with Ni-NTA His·Bind Resin (Novagen), loaded into a column, and the flow-through was discarded. The column was washed twice with Wash Buffer (50 mM NaH_2_PO_4_, pH 8.0, 300 mM NaCl, 20 mM imidazole) and the recombinant proteins were eluted with Elution buffer (50 mM NaH_2_PO_4_, pH 8.0, 300 mM NaCl, 250 mM imidazole) in four fractions, which were analyzed by SDS-PAGE. Purified recombinant proteins were incubated at concentrations of 0.18, 0.95 and 0.53 mg/ml of protein for gas1p, gas2p and gas5p, respectively, with 4 mM of reduced laminari-oligosaccharides (G_13r_ or G_20r_) in 20 µl of 50 mM NaOAc, pH 5.0, at 37°C. Sequential aliquots of 2.5 µl supplemented with 40 µl of 50 mM NaOH were analyzed by HPAEC with a CarboPAC-PA200 column (Dionex, 3.2×250 mm), as previously described [39].

## Supporting Information

Figure S1 Characteristics of *S. pombe* GH72 proteins. (A) Summary of the main characteristics of *S. pombe* GH72 proteins. (B) gas1p, gas4p and gas5p contain a hydrophobic signal at the C-termini for GPI-attachment. Hydrophobicity profile of gas1p, gas2p, gas4p and gas5p. The hydrophobic regions at the C-termini are highlighted in grey. To the right, the cleavage points for GPI attachment proposed by GPI-SOM are shown by an arrowhead.
(0.18 MB TIF)

Figure S2 Time-lapse microscopic analysis of the *gas1*Δ mutant. *gas1*Δ cells grown in liquid media with osmotic support were inoculated on YES solid medium and observed under a microscope equipped for Nomarski optics. Images were captured every minute. Growth, polarity defects and cell lysis along the time are shown. Black arrowheads point to cell lysis at each time point. Asterisks highlight cells with defects in polarity. Scale bars, 10 μm.
(1.08 MB TIF)

Figure S3 (A) β(1,3)-glucanosyl-transferase activity of recombinant gas1p, gas2p and gas5p using G13r as substrate. Reactions were incubated in 50 mM acetate buffer (pH 5.0) for the indicated times. The reaction products were analyzed by HPAEC-PED on a CarboPAC-PA200 column. (B) *S. pombe* cell walls were extracted with 1 M NaOH to separate alkali-soluble materials (AS) from the alkali-insoluble (AI) by centrifugation. The amount of each fraction was estimated by colorimetric assays of total proteins and hexoses. The percentage corresponds to the fraction of hexoses in each fraction relative to the total amount of hexoses detected in the dried cell walls. (C) Gel filtration chromatography of carboxymethylated cell wall fractions on an HR500S column. An aliquot of the different fractions was digested by Laminarinase-A before the carboxymethylation and was compared with the untreated control. Sugars were detected with the phenol-sulphuric assay.
(0.16 MB TIF)

Figure S4 Over-expression of gas^+^ genes complements the defects of the *S. pombe gas1*Δ mutant. (A) Growth rate of gas1^+^ strain carrying the vector (YMMR135) and *gas1*Δ cells harbouring vector (YMMR133), Pnmt1-gas2^+^ (YMMR138) or Pnmt1-gas5^+^ (YMMR139), in media with (right) and without (left) sorbitol. (B) Microscopic appearance of gas1Δ cells ectopically expressing gas2^+^ or gas5^+^. Wild-type cells and *gas1*Δ strains harbouring the different constructs were incubated in minimal media with or without osmotic support. Samples were stained directly with aniline blue before images were captured. Differential interference contrast (DIC) or fluorescence (AB) photographs are shown. Scale bars, 10 μm.
(1.73 MB TIF)
