# Supplementary figures and images for: β(1,3)-Glucanosyl-Transferase Activity Is Essential for Cell Wall Integrity and Viability of Schizosaccharomyces pombe
Source: PLoS One. 2010 Nov 18;5(11):e14046. doi: 10.1371/journal.pone.0014046 (PMC2987803; doi:10.1371/journal.pone.0014046)

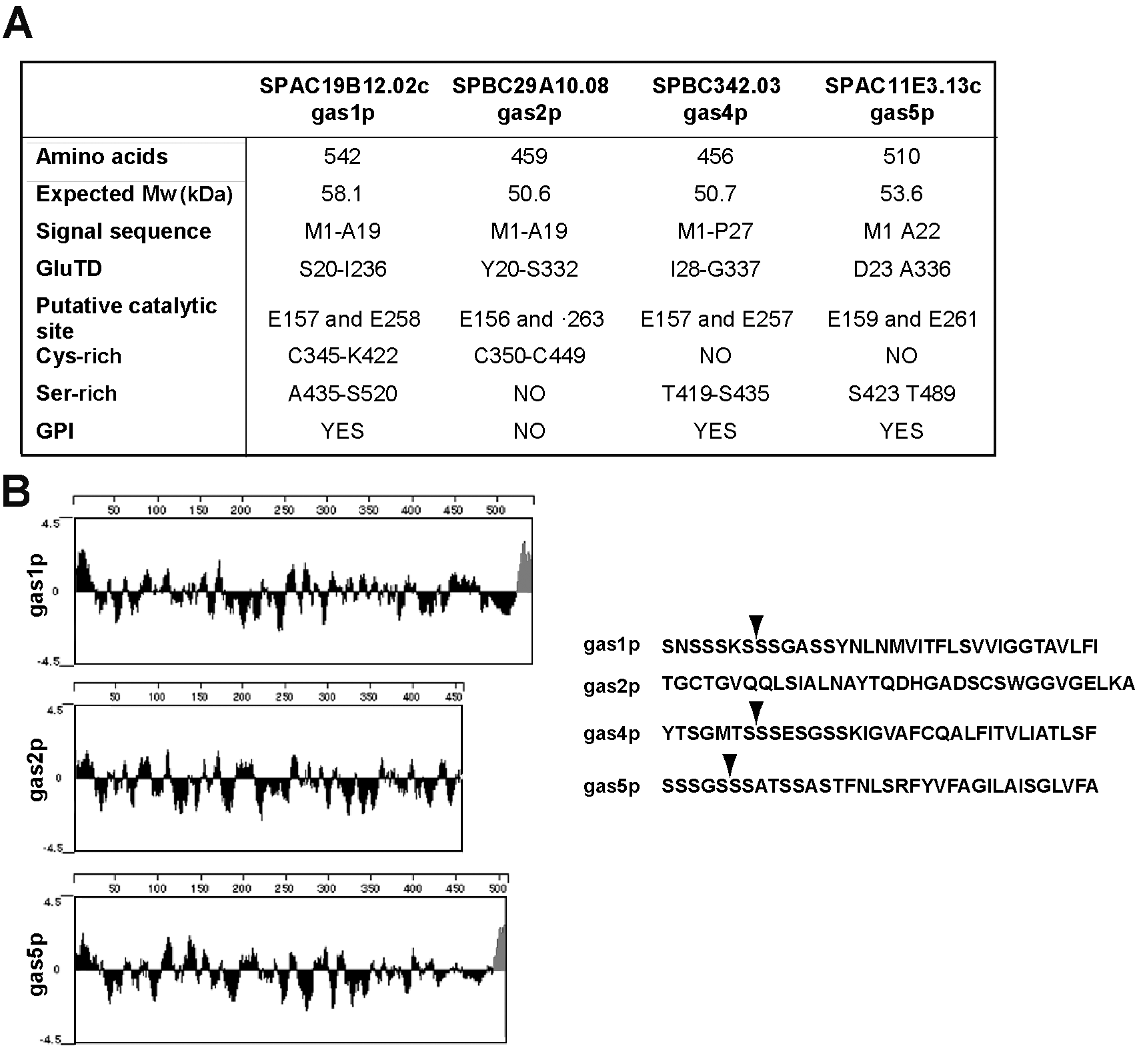

Supplement: Figure S1 — Characteristics of S. pombe GH72 proteins. (A) Summary of the main characteristics of S. pombe GH72 proteins. (B) gas1p, gas4p and gas5p contain a hydrophobic signal at the C-termini for GPI-attachment. Hydrophobicity profile of gas1p, gas2p, gas4p and gas5p. The hydrophobic regions at the C-termini are highlighted in grey. To the right, the cleavage points for GPI attachment proposed by GPI-SOM are shown by an arrowhead. (0.18 MB TIF) [file pone.0014046.s001.tif]

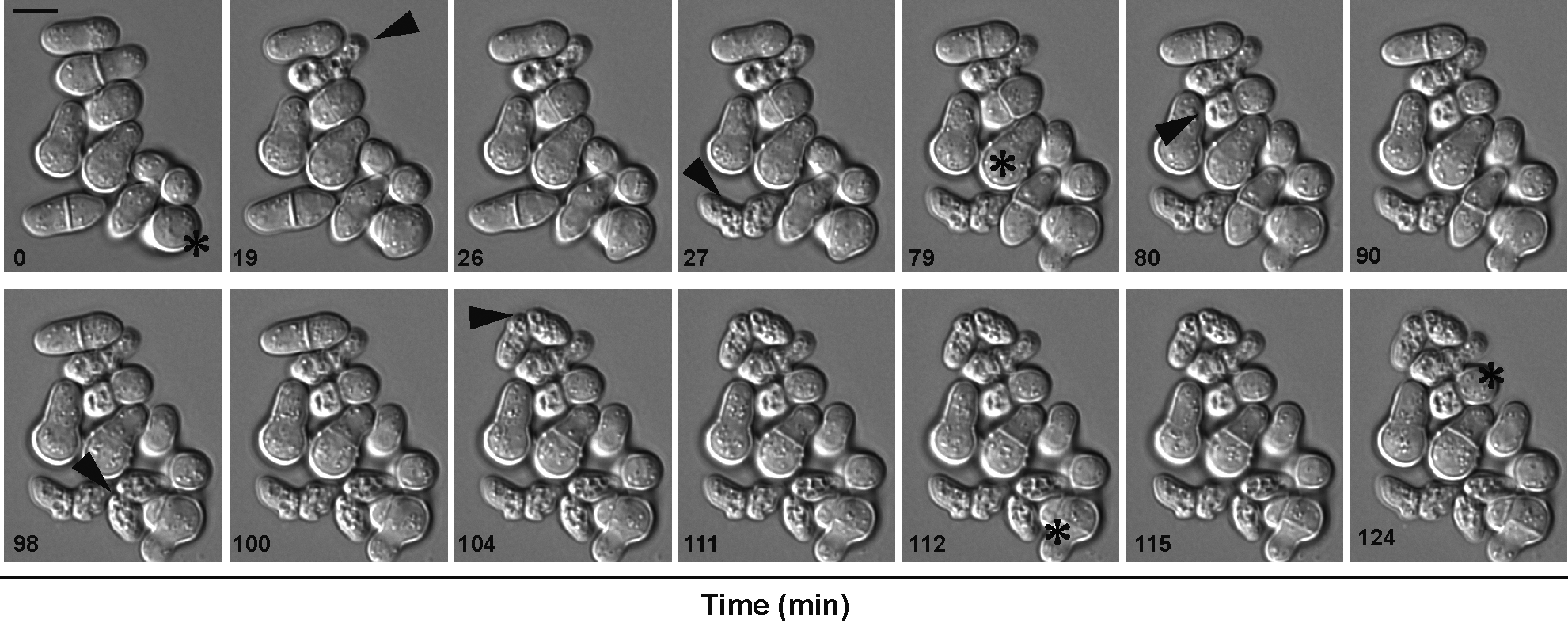

Supplement: Figure S2 — Time-lapse microscopic analysis of the gas1Δ mutant. gas1Δ cells grown in liquid media with osmotic support were inoculated on YES solid medium and observed under a microscope equipped for Nomarski optics. Images were captured every minute. Growth, polarity defects and cell lysis along the time are shown. Black arrowheads point to cell lysis at each time point. Asterisks highlight cells with defects in polarity. Scale bars, 10 μm. (1.08 MB TIF) [file pone.0014046.s002.tif]

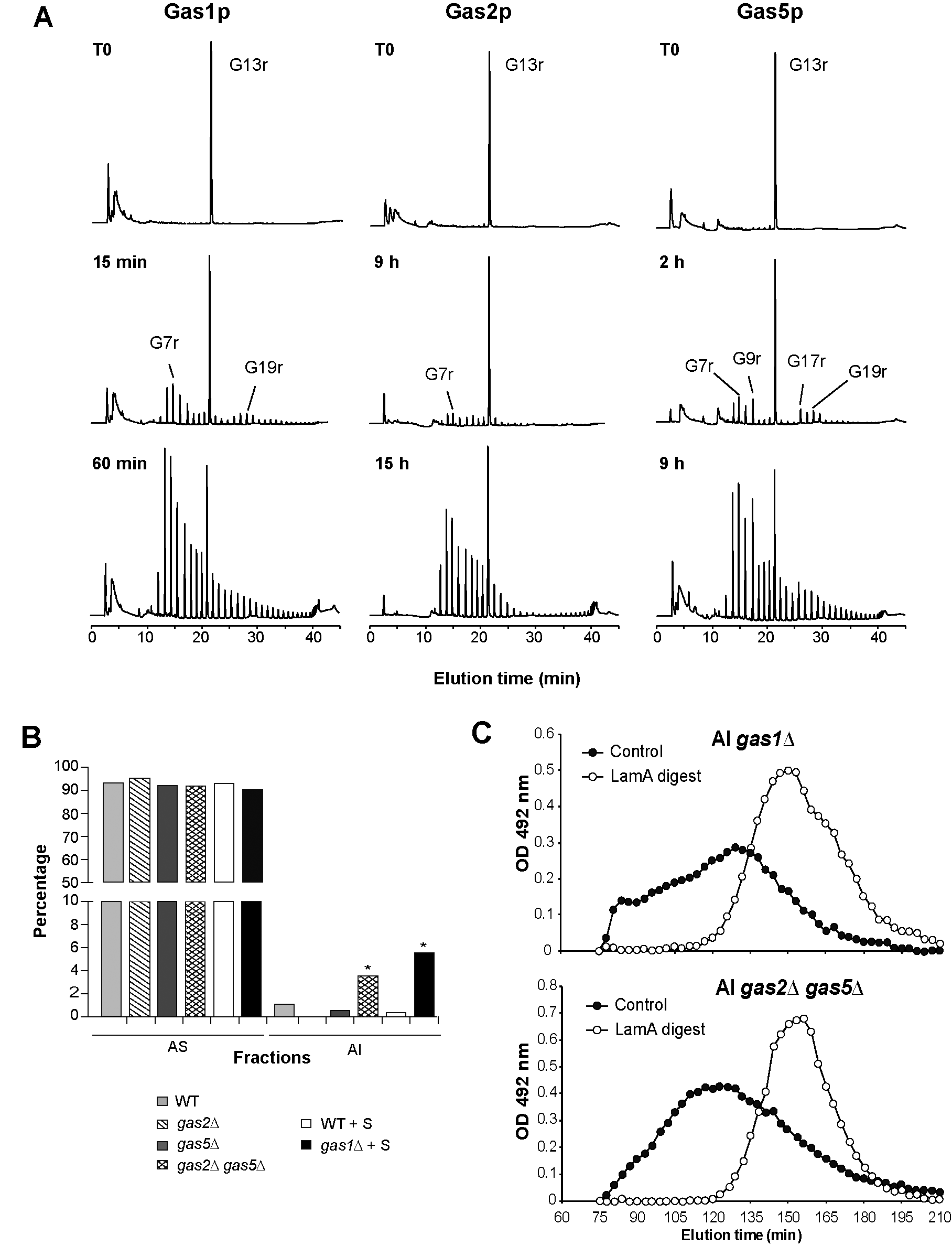

Supplement: Figure S3 — (A) β(1,3)-glucanosyl-transferase activity of recombinant gas1p, gas2p and gas5p using G13r as substrate. Reactions were incubated in 50 mM acetate buffer (pH 5.0) for the indicated times. The reaction products were analyzed by HPAEC-PED on a CarboPAC-PA200 column. (B) S. pombe cell walls were extracted with 1 M NaOH to separate alkali-soluble materials (AS) from the alkali-insoluble (AI) by centrifugation. The amount of each fraction was estimated by colorimetric assays of total proteins and hexoses. The percentage corresponds to the fraction of hexoses in each fraction relative to the total amount of hexoses detected in the dried cell walls. (C) Gel filtration chromatography of carboxymethylated cell wall fractions on an HR500S column. An aliquot of the different fractions was digested by Laminarinase-A before the carboxymethylation and was compared with the untreated control. Sugars were detected with the phenol-sulphuric assay. (0.16 MB TIF) [file pone.0014046.s003.tif]

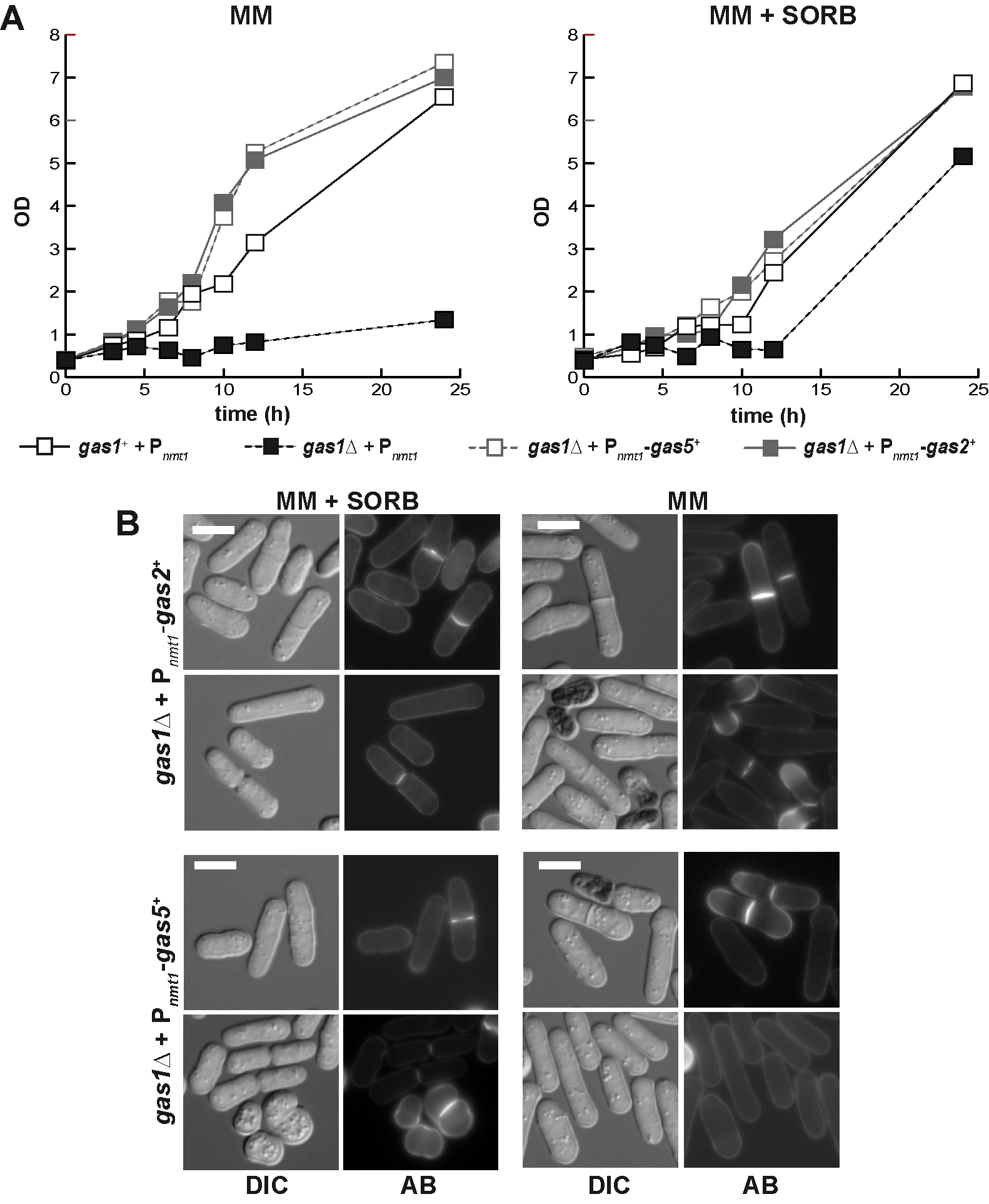

Supplement: Figure S4 — Over-expression of gas+ genes complements the defects of the S. pombe gas1Δ mutant. (A) Growth rate of gas1+ strain carrying the vector (YMMR135) and gas1Δ cells harbouring vector (YMMR133), Pnmt1-gas2+ (YMMR138) or Pnmt1-gas5+ (YMMR139), in media with (right) and without (left) sorbitol. (B) Microscopic appearance of gas1Δ cells ectopically expressing gas2+ or gas5+. Wild-type cells and gas1Δ strains harbouring the different constructs were incubated in minimal media with or without osmotic support. Samples were stained directly with aniline blue before images were captured. Differential interference contrast (DIC) or fluorescence (AB) photographs are shown. Scale bars, 10 μm. (1.73 MB TIF) [file pone.0014046.s004.tif]
